# 2739. Evaluation of a Perioperative Fungal Prophylaxis Protocol Change in Orthotopic Liver Transplant

**DOI:** 10.1093/ofid/ofad500.2350

**Published:** 2023-11-27

**Authors:** Megan Shulkosky, Xhilda Xhemali, Jamie Eckardt, Jessica Ward, Heather Schlick, Kyle D Brizendine, Nicole Palm

**Affiliations:** ProMedica Toledo Hospital; Cleveland Clinic, Cleveland, Ohio; Cleveland Clinic, Cleveland, Ohio; Cleveland Clinic, Cleveland, Ohio; Cleveland Clinic, Cleveland, Ohio; Cleveland Clinic Foundation, Cleveland, OH; Cleveland Clinic, Cleveland, Ohio

## Abstract

**Background:**

Orthotopic liver transplant (OLT) carries a high rate of fungal infections, including invasive fungal infections (IFI). A targeted antifungal prophylaxis protocol can identify OLT patients who warrant antifungal prophylaxis and optimize agent selection. In January 2021, a targeted antifungal prophylaxis post-liver transplant protocol was implemented at Cleveland Clinic. Since its introduction, rates of fungal infections and adherence to the protocol have not been assessed. The goal of this project is to describe the impact of a protocol change in OLT recipients.

**Figure:**
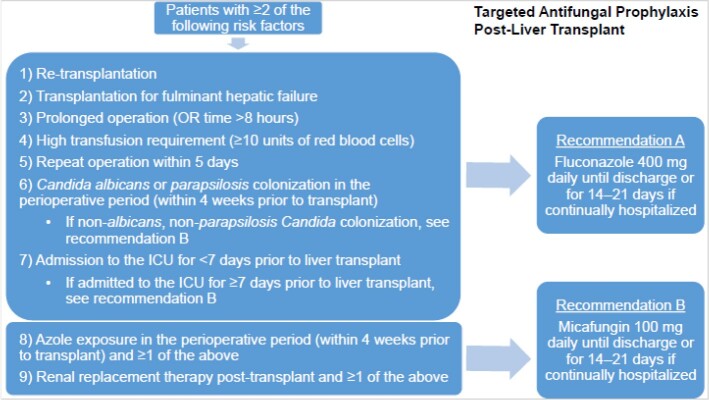

**Methods:**

This was a retrospective observational cohort study of patients ≥ 18 years old with an OLT +/- sequential kidney transplant at Cleveland Clinic. Review of perioperative fungal prophylaxis from June 1, 2019 – May 31, 2020 was performed and used as the baseline. The post-intervention period was June 1, 2021 – May 31, 2022. The primary objective was to determine adherence to the new OLT fungal prophylaxis protocol on post-operative day (POD) 0. Secondary objectives included the 90-day incidence of proven or probable IFI post-OLT before and after implementation.

Study Population

**Figure:**
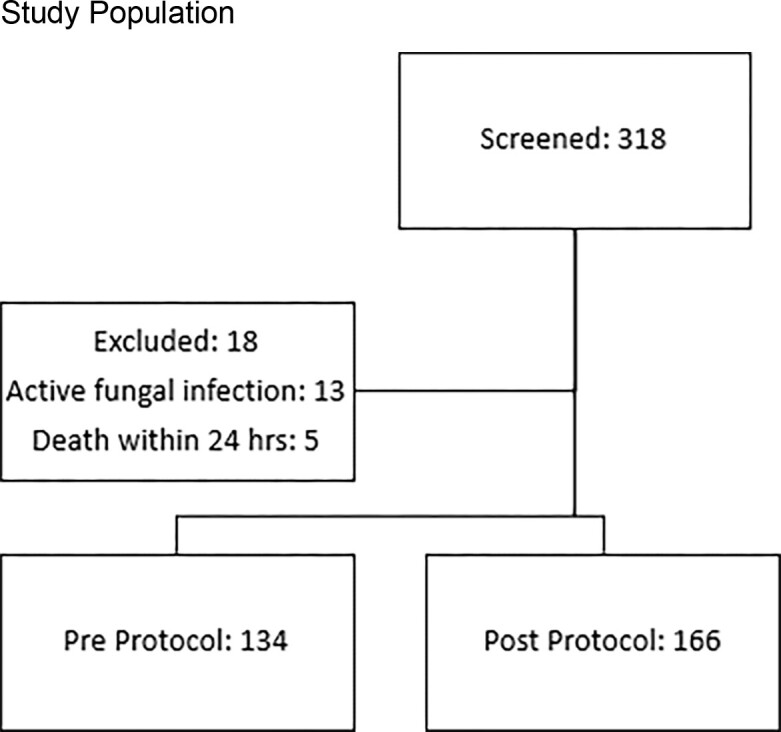

**Results:**

A total of 134 patients pre-protocol and 166 patients post-protocol were included. Prior to protocol implementation, 73% patients were prescribed clotrimazole, 13% fluconazole, 13% micafungin, and 1% nystatin. After protocol implementation, 63% were prescribed clotrimazole, 16% fluconazole, and 21% micafungin. In the post-protocol group, there was an adherence rate of 66% on POD0 which increased to 84% over the duration of prophylaxis. Prior to initiation of the antifungal protocol, 6.7% of patients developed an IFI while 3.6% of patients developed an IFI post-protocol (p=0.22). Median time to IFI was 8 days (IQR 2-19) pre-protocol and 15 days (IQR 6-17) post-protocol. Mortality and ICU length of stay were comparable between groups.

Primary and Secondary Results

**Figure:**
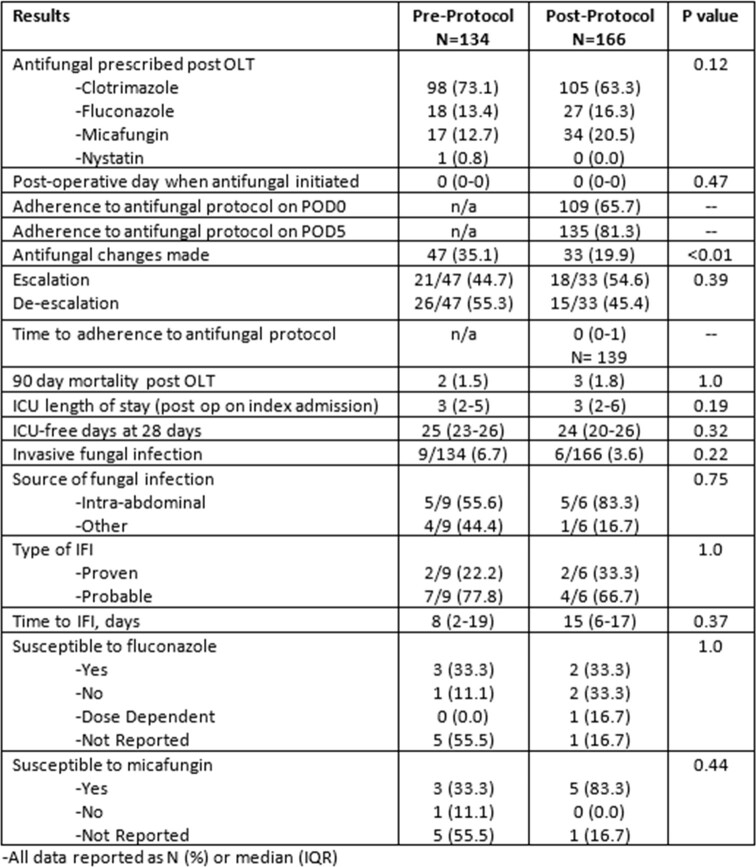

OLT: orthotopic liver transplant; POD: post-operative day; ICU: intensive care unit; IFI: invasive fungal infection

Characteristics of Patients with Invasive Fungal Infections

**Figure:**
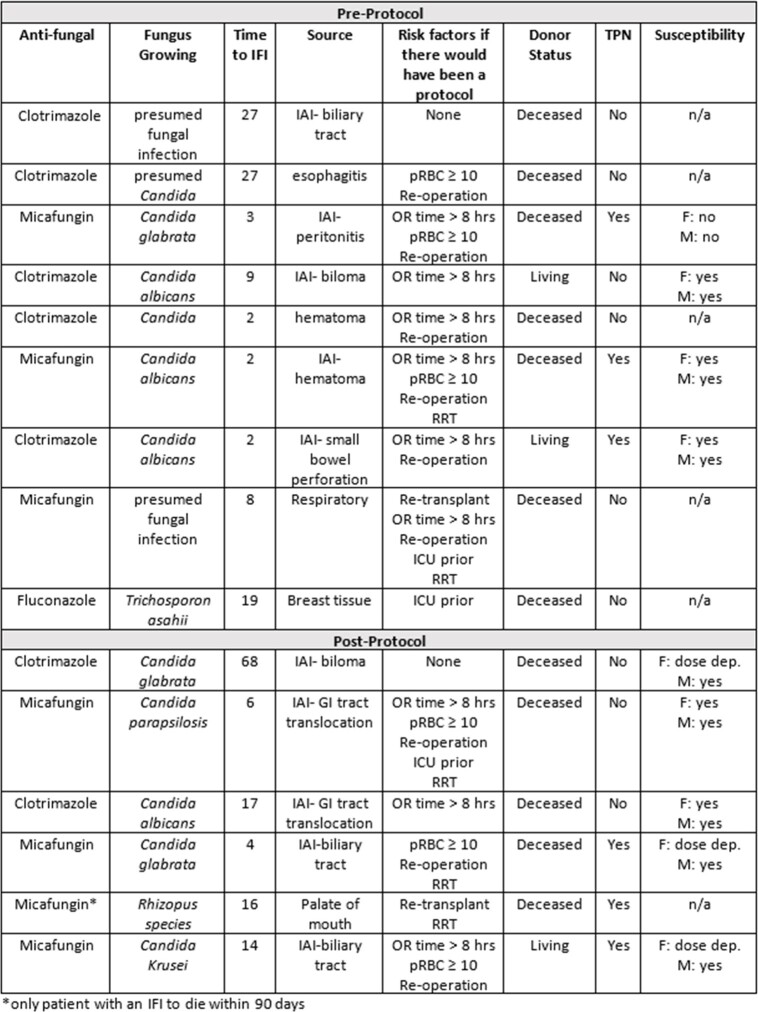

IFI: invasive fungal infection; TPN: total parenteral nutrition; IAI: intra-abdominal infection; n/a: not applicable; pRBC: packed red blood cells; OR: operating room; RRT: renal replacement therapy; ICU: intensive care unit; Dose dep.: dose dependent; GI: gastrointestinal

**Conclusion:**

The implementation of a targeted antifungal prophylaxis post-liver transplant protocol can be a powerful strategy for promoting consistency in antifungal prophylaxis within a population at high risk for IFIs. Our study showed an 84% adherence rate to the implemented protocol, with numerically lower rates of IFIs post-protocol compared to pre-protocol.

**Disclosures:**

**All Authors**: No reported disclosures

